# Editorial Changes

**Published:** 1849-03

**Authors:** 


### EDITORIAL CHANGES.

Professor Huston, who has conducted the Medical Examiner with so much independence and ability for a number of years, has dissolved his connection with that Journal, and is succeeded by Francis G. Smith, M.D., and David H. Tucker, M.D. The reputation of his successors is a guaranty that the Examiner will maintain its high rank among its cotemporaries, but we are sorry, nevertheless, to part with Professor Huston. He is an able critic, and has always been ready to give his opinions upon the topics and things coming before him This boldness has often brought him into conflict with his professional brethren, but in all his controversies he has evinced courtesy and a love of truth; and in retiring we are sure he carries with him the regrets and good wishes of his readers, and the respect of the profession.

We copy the following from the Buffalo Medical Journal:

"By a copy of the Cleveland Daily Herald, which has been politely forwarded, we perceive that the first number of a new medical journal, entitled the Western Medical Magazine and Review, is to be issued on the first of the present month. It is to be edited by J. J. Delamater, M.D., Professor of Anatomy and Physiology in the Cleveland Medical College, assisted by his colleagues as collaborators. It is to be a bi-monthly of 128 pages. We trust that the editor may find in his editorial duties, an agreeable relaxation from the labors and cares incident to his prefessorial and professional avocations. With such an expectation probably many undertakings of the kind have been commenced; how fully it may be realized, experience will reveal to Dr. D., as it has to those who have anticipated him in a similar experiment. We heartily wish the effort success, extending, as in courtesy bound, to a newly acquired brother of the *corps editoriale,* the hand of fraternal fellowship.

"The able editor of the Southern Medical and Surgical Journal, Professor Paul F. Eve, has given notice that the Journal will be discontinued after the present year, owing to want of adequate patronage. He states that the privilege of laboring for his readers during the past year, has required from his own pocket, an outlay of $900, and he very reasonably concludes that the luxury of serving the medical public on these terms is too expensive to be longer indulged. That a periodical of such merit as the Southern Medical and Surgical Journal should require more than the gratuitous services of the editor to secure ample support, is a stigma upon our southern brethren which we sincerely hope they will not consent to bear. We anticipate that the effect of the notice referred to, will be to secure a list of *paying* subscribers, that will not only justify the continuance of the work, but furnish a complimentary testimonial to the value of the editor's past labors."

We cordially adopt these sentiments. We hope and believe better things of the physicians of the South, than that they will suffer a journal so faithfully edited as that of which Professor Eve has had charge, to fail for want of pecuniary support."

